# Synthesis of Fe_3_O_4_@MCM-48 as Nano Fertilizer for Growth Stimulation in Tomato Plants

**DOI:** 10.3390/plants14030405

**Published:** 2025-01-29

**Authors:** Adriana Morfín-Gutiérrez, Luis Alfonso García-Cerda, Yolanda González-García, Antonio Juárez-Maldonado

**Affiliations:** 1 Departamento de Botánica, Universidad Autónoma Agraria Antonio Narro, Calz Antonio Narro 1923, Buenavista, Saltillo 25315, Mexico; adrianamorgut@gmail.com; 2 Centro de Investigación en Química Aplicada, Blvd. Enrique Reyna Hermosillo #140, Saltillo 25294, Mexico; luis.garcia@ciqa.edu.mx; 3 Instituto Nacional de Investigaciones Forestales, Agrícolas y Pecuarias, Centro de Investigación Regional Noroeste, Campo Experimental Todos Santos, La Paz 23070, Mexico; yolanda_glezg@hotmail.com; 4 Laboratorio Nacional Conahcyt de Ecofisiología Vegetal y Seguridad Alimentaria (LANCEVSA), Universidad Autónoma Agraria Antonio Narro, Saltillo 25315, Mexico

**Keywords:** crop growth, fruit quality, magnetite, mineral deficiency stress, nanoparticles

## Abstract

Innovative nano fertilizers based on nanoparticles present great potential for agriculture since they can stimulate growth and development in different crops. However, the efficiency of nanoparticles directly depends on their physicochemical characteristics, such as composition, shape, size, and the type of plant species. In this work, a material formed by mesoporous silica and iron oxide (Fe_3_O_4_@MCM-48) was synthesized and used as a nano fertilizer for tomato crop. Materials with different percentages of iron (10, 20, 30, 40, and 50% by weight) were applied to study the effect of the amount of iron in the plants and compared with MCM-48 without iron and ferric chloride hexahydrate. Using X-ray diffraction (XRD), it was possible to identify the phases present in the system, and with Transmission Electron Microscopy (TEM), it was observed that the material is made up of a matrix of MCM-48 with embedded Fe_3_O_4_ nanoparticles with a size of 5 nm. Also, the results show that all treatments with nano fertilizers increased the content of photosynthetic pigments and carotenoids in leaves. The use of nano fertilizers can be a viable option to improve the crop growth and efficiency of nutrient use in plants.

## 1. Introduction

Due to population increase and the great demand for food, fertilizers are necessary to increase agricultural productivity. However, the absorption efficiency in plants is very low due to the conversion of nutrients into their insoluble form, with fertilization effectiveness of 20–50%, particularly elements such as nitrogen, phosphorus, and potassium reporting values of 30–60%, 10–20%, and 35–40%, respectively [1,2]. Nutrient losses affect crop yields and cause severe problems with water, air, and soil pollution [3].

Using nanoparticles as nutrient-carrying systems in an available form is an alternative to improve absorption through plants. This type of nano fertilizer (NF) presents some relevant characteristics, such as precise doses of nutrients through foliar and root applications, high fertilization efficiency, and minimized contamination problems [4]. Regarding the production costs of NF, there is still little clarity and there is still a lack of comparison with the production costs of conventional fertilizers [5]. However, considering the potential benefits of NF in crops, as well as the higher nutrient use efficiency and therefore lower application quantity, it may be economically feasible to use NF in agriculture.

Nanotechnology is currently a science that plays an important role in agriculture because it allows the synthesis of nanoparticles with desired characteristics and properties, which are part of new high-quality nano-based products that can be used as growth stimulants in various crops [6]. Nanoparticles can be used in high-precision agriculture; that is, they serve as fertilizers in crops, improving the dosage of nutrients and minimizing the loss of fertilizers, which results in a decrease in environmental pollution caused by the uncontrolled use of agrochemicals [7].

Silica is a mineral widely available naturally. However, the discovery of mesoporous silica makes it an important material due to its interesting applications as a release and absorption system and its biocompatibility, thermal stability, and non-toxicity. On the other hand, its synthesis is simple and low cost; furthermore, adjusting the experimental conditions makes it possible to modify the pore size, pore volume, mesostructure ordering, and particle shape. One of the different configurations of silica is MCM-48 (Mobil Crystalline Materials), which has a pore size between 2 and 50 nm with a cubic pore arrangement [8,9].

According to some investigations, silica is a material with great potential in agriculture, used as a nano pesticide, nano herbicide, and nano fertilizer. It helps plants improve their tolerance to biotic and abiotic stress, directly impacting crop growth and development. Moreover, it acts as a protective agent against fungal and bacterial infections [6,10,11].

Iron is an essential micronutrient for plants, with some fundamental functions in plants, like regulating growth and development and participating in cellular processes (chlorophyll biosynthesis, photosynthesis, etc.) [12]. Therefore, a deficiency of this element provokes changes in the structure and functioning of plants. An example of iron deficiency is iron chlorosis in plant crops and fruit trees on calcareous soils, which consists of a nutritional disorder that becomes evident with a yellowish color in the upper leaves of a plant, as well as in the delay of its growth, which reduces the fruit yield and/or fruit quality of many crops. These types of disorders are a consequence of the low availability of iron in alkaline soils and defective mechanisms of iron absorption and utilization, altering the metabolism of plants [13,14]. To solve this problem, foliar applications of FeSO_4_, Fe ethylenediamine tetra-acetic acid (Fe-EDTA), or Fe–ethylene diamine-N, N’-bis (hydroxy phenyl) acetic acid are made. However, in some cases, they can be costly and highly polluting compounds [15]. Therefore, an iron administration in nanoparticle form could improve the iron availability for plants [12], their stress tolerance, and their nutrient uptake [16].

An advantage of using NF is the lesser impact on the environment, thus increasing nutrient efficiency, in addition to the beneficial effect of stimulating crop productivity. Other beneficial characteristics of NF are higher nutrient absorption efficiency, controlled release, higher bioavailability, and higher solubility [3,5,17]. In addition, one of the great challenges facing agriculture today is the negative impact of environmental conditions on crops, as it is increasingly common to observe stressful conditions for plants such as water and soil stress. NFs are an option to face different environmental stresses and improve crop productivity [5,17]. In the case of Fe-based NFs, there are sufficient data demonstrating their positive impacts compared to conventional fertilizers on a variety of crops. These include improved plant growth; increased productivity; improved physiological parameters such as photosynthesis, and respiration; induction of stress tolerance; improved antioxidant status; and increased Fe content, among others [18]. However, most of the works only evaluate Fe nanoparticles in different concentrations or Fe-based nanocomposites with a single concentration of the element [17,18,19]. In relation to the induction of stress tolerance, it has also been demonstrated that the use of different iron-based nanomaterials (α-Fe_2_O_3_ NMs, γ-Fe_2_O_3_ NMs, and Fe_3_O_4_ NMs, at 50 mg Fe L^−1^) have been effective in reducing the negative impact of cadmium on wheat crops, by decreasing the concentration of this element in plant tissues and improving productivity [20].

According to some research, MCM-48 is a nanomaterial with a three-dimensional pore structure [21]. It is commonly used for absorption, loading, and release of various compounds; since its pores function as reservoirs and through diffusion, a release of them is possible [22,23,24,25]. Considering this, the Fe_3_O_4_@MCM-48 system can be an alternative in crop fertilization. It will allow a slow release of magnetite nanoparticles through diffusion, leaving iron available only for plants. This research aimed to synthesize an MCM-48 carrier with different percentages of iron to stimulate growth and development in tomato plants.

## 2. Results

### 2.1. Characteristics of Fe_3_O_4_@MCM-48

**Figure 1:**
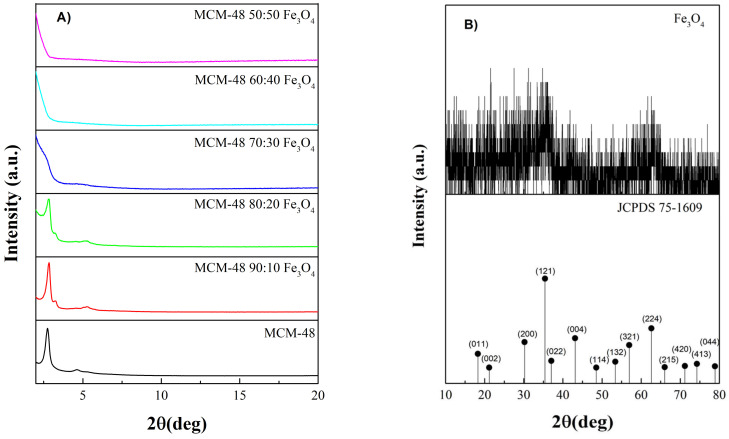
XRD patterns of (**A**) MCM-48 silica calcined at 540 °C and synthesized at different Fe_3_O_4_ concentrations, (**B**) MCM-48 50:50 Fe_3_O_4_ calcined at 800 °C.

XRD was used to obtain information about the structural changes of the unmodified MCM-48 and MCM-48 with different contents of Fe_3_O_4_ nanoparticles (Figure 1A). The diffractogram of pristine MCM-48 shows reflections at 2.8 and 4.5°, corresponding to the (211) and (321) planes. While the diffractograms of MCM-48 with the incorporation of 10 and 20% of Fe_3_O_4_, diffraction peaks are observed at 2.8, 3.2, and 5.2° corresponding to the planes (211), (220), and (332), respectively [20]. Additionally, Figure 1A did not present diffraction peaks that would demonstrate the presence of the nanoparticles incorporated into the MCM-48. Due to the above, it was necessary to increase the particle size by calcining at 800 °C (MCM-48 50:50 Fe_3_O_4_), and later, it was possible to observe the characteristic peaks (Figure 1B) corresponding to magnetite according to the reference JCPDS 75-1609.

The amount of iron in the MCM-48 for the different nanocomposites was quantified using the inductively coupled plasma atomic emission spectroscopy (ICP-OES) analysis. Table 1 shows the amounts of magnetite available for absorption by tomato plants.

**Figure 2:**
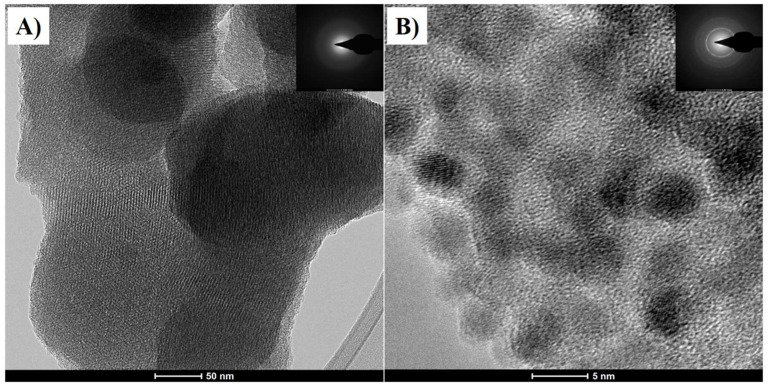

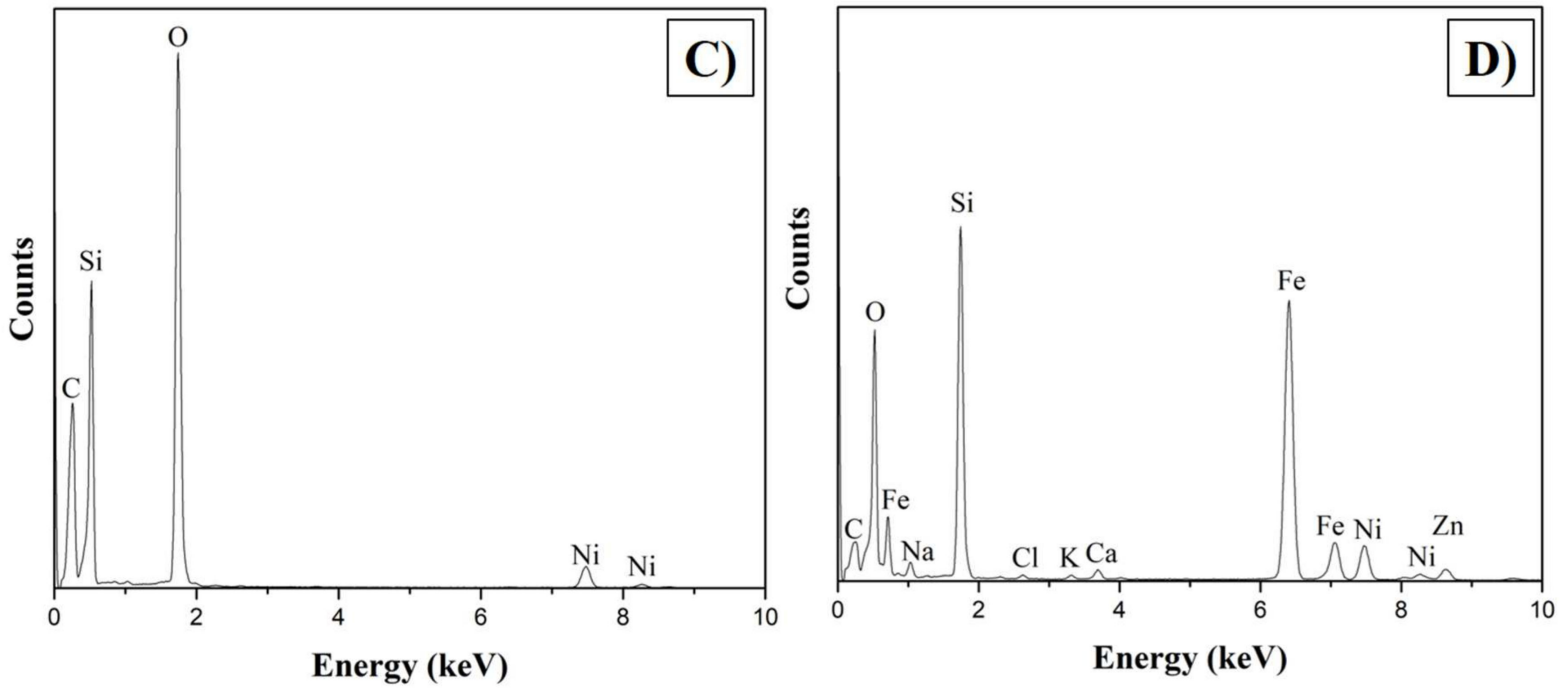
TEM images for MCM-48 (**A**), MCM-48 60:40 Fe_3_O_4_ (**B**), and EDS analysis, respectively (**C**,**D**).

High-resolution TEM was used to analyze the microstructure of MCM-48 before and after incorporating the magnetic nanoparticles. Figure 2A shows the unmodified micrograph of MCM-48, and the energy-dispersive X-ray spectroscopy (EDS) analysis (Figure 2C) presents the chemical composition. Figure 2B shows the micrograph of MCM-48 with 40% Fe_3_O_4_ and EDS analysis (Figure 2D) from the nanocomposite. Finally, an amorphous material for the image MCM-48 was observed through the analysis by selected area electron diffraction (SAED) (inset in Figure 2A). In contrast, the image for MCM-48 60:40 Fe_3_O_4_ presents crystalline nanoparticles with spherical morphology (Figure 2B).

### 2.2. Agronomical Parameters

**Figure 3:**
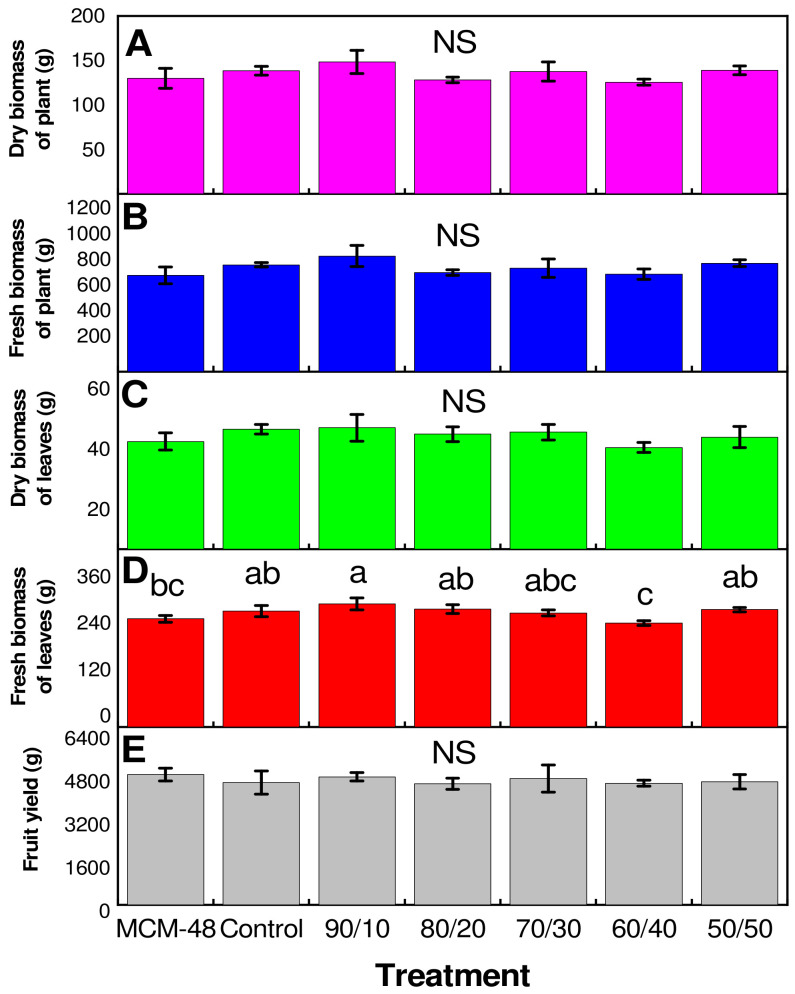
Impact of MCM-48 with different concentrations of Fe_3_O_4_ on (**A**) dry biomass of plants, (**B**) fresh biomass of plants, (**C**) dry biomass of leaves, (**D**) fresh biomass of leaves, and (**E**) fruit yield. n = 3 ± standard error. NS = Not significant. According to Fisher’s least significant difference (*p* < 0.05), different letters indicate significant differences between treatments.

Figure 3 shows the agrochemical parameters for the different treatments, where the results indicate that fresh biomass in plants presents differences between treatments only (Figure 3D, Table S1). In fresh biomass of leaves, the 90/10 treatment was better than the other treatments (Figure 3D). The dry and fresh biomass of plant and dry biomass of leaves consistently showed that the 90/10 treatment presented the best result, however, it was not statistically different from the other treatments.

### 2.3. Biochemical Variables

**Figure 4:**
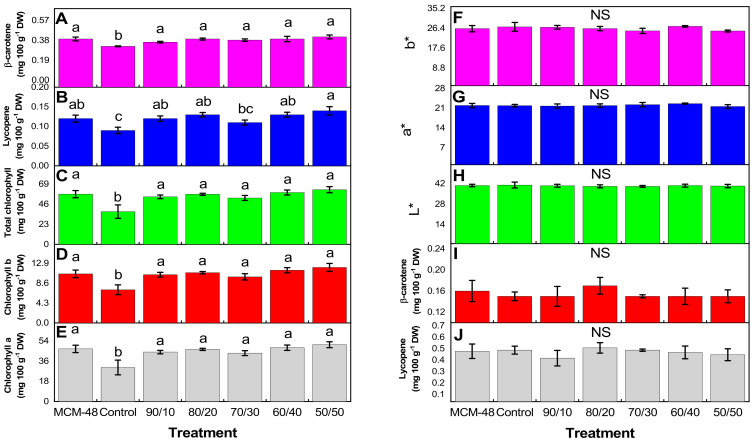
Content of photosynthetic pigments (**A**) β-carotene, (**B**) lycopene, (**C**) total chlorophylls, (**D**) chlorophyll b, and (**E**) chlorophyll a, in leaves of tomato plants exposed to different treatments with nano fertilizers. Colorimetric parameters (**F**) b*, (**G**) a*, (**H**) L*, and photosynthetic pigments (**I**) β-carotene and (**J**) lycopene in fruits. n = 3 ± standard error. NS = Not significant. According to Fisher’s least significant difference (*p* < 0.05), different letters indicate significant differences between treatments.

The results of the photosynthetic pigments of leaves and tomato fruits of crops exposed to the different nanofertilizer treatments are shown in Figure 4 (Table S2). In the case of chlorophylls and carotenoids in leaves (Figure 4A–E), it is generally observed that an increase in the same samples is treated with the different compositions of nanomaterials, except for the 70/30 treatment for lycopene (Figure 4B). The increases observed for β-carotene were between 10.3 and 23.1%, for lycopene 33.33 and 55.55%, for total chlorophyll between 41.54 and 66.93%, for chlorophyll *b* 38.99 and 67.40%, and chlorophyll *a* 42.14 and 66.82%, with respect to the control. Carotenoids and color parameters in fruits (Figure 4F–J) did not present statistically significant differences.

### 2.4. Physiochemical Parameters of Fruits

**Figure 5:**
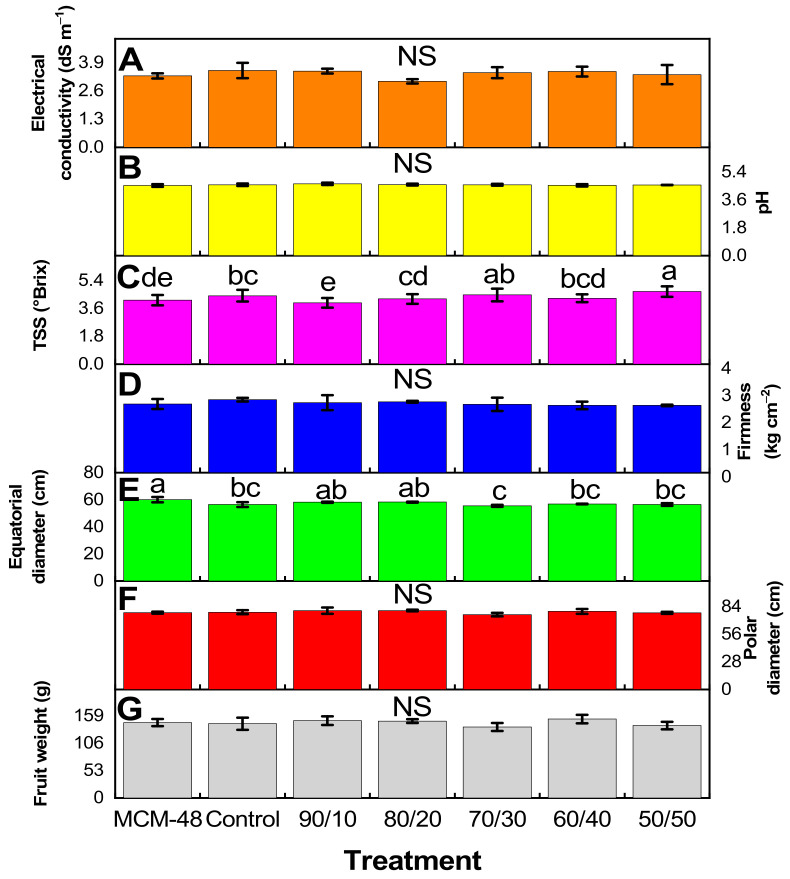
Physico-chemical parameters, (**A**) electrical conductivity, (**B**) pH, (**C**) TSS, (**D**) Firmness, (**E**) Equatorial diameter, (**F**) polar diameter, and (**G**) fruit weight for tomato fruits. n = 3 ± standard error. NS = Not significant. Different letters indicate significant differences between treatments according to Fisher’s least significant difference (*p* < 0.05).

The results of the physicochemical analyses shown in Figure 5 were carried out on fruits with the same maturity and commercial quality (Table S3). Electrical conductivity, pH, firmness, polar diameter, and fruit weight do not present statistically significant differences in Figure 5A, Figure 5B, Figure 5D, Figure 5F and Figure 5G, respectively. In the case of total soluble solids (Figure 5C), differences were observed between the 90/10 and 50/50 treatments. In the equatorial diameter (Figure 5E), it was observed that the MCM-48 treatment was better than the 70/30 treatment.

### 2.5. ICP-OES Analysis on Leaves

**Figure 6:**
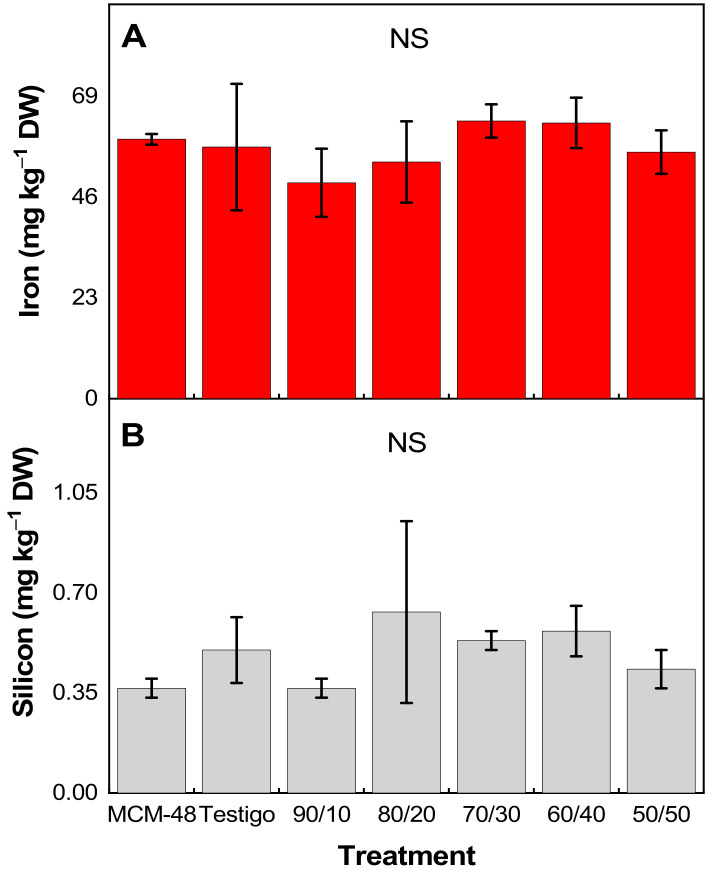
Accumulation of (**A**) iron and (**B**) silicon in leaves of tomato plants. n = 3 ± standard error.

Through the ICP-OES analysis, it was possible to quantify the Fe_3_O_4_ (Figure 6A) and MCM-48 (Figure 6B) present in tomato leaves treated with different concentrations of nano fertilizer. The results did not present statistically significant differences between treatments (Table S4).

## 3. Discussion

### 3.1. Synthesis and Characterization of Fe_3_O_4_@MCM-48

From the XRD results, it is observed that, as the iron content increases, the diffraction peaks of MCM-48 disappear due to a possible gradual loss of long-range ordering when doped with Fe_3_O_4_ [26,27]. Additionally, Figure 1A did not present diffraction peaks that would demonstrate the presence of the nanoparticles incorporated into the MCM-48, which could be because the Fe_3_O_4_ were uniformly doped into the MCM-48 framework [28]. Due to the above, it was necessary to increase the particle size by calcining at 800 °C (MCM-48 50:50 Fe_3_O_4_), and later, it was possible to observe the characteristic peaks (Figure 1B) corresponding to magnetite according to the reference JCPDS 75-1609.

From high-resolution transmission electron microscopy images, it is observed that the unmodified MCM-48 presents a well-organized structure throughout the entire sample with a uniform pore system that coincides with the images reported for MCM-48 [29,30,31] and, according to the EDS analysis (Figure 2C), the sample presents silicon and oxygen. On the other hand, Figure 2B shows the micrograph of MCM-48 with 40% Fe_3_O_4_, where Fe_3_O_4_ nanoparticles of approximately 5 nm embedded in a silica matrix can be seen, and its EDS analysis (Figure 2D), allows identifying the presence of Fe, O, and Si, as well as small traces of impurity elements. Finally, MCM-48 shows an amorphous material (Figure 2A), while for MCM-48 Fe_3_O_4_ crystalline nanoparticles with spherical morphology were observed (Figure 2B).

Mesoporous materials have recently been used as delivery agents in the loading and release of different compounds since they present interesting characteristics such as thermal stability, biocompatibility, an ordered porosity with homogeneous sizes that allow the loading and controlled release of compounds, in addition to pores with a large volume for housing materials and their high surface area that allows them to be absorbed easily [32]. On the other hand, Fe is one of the essential micronutrients for the growth and development of crops. However, its efficiency is limited by its low availability in alkaline soils and the formation of various insoluble complexes for plants. Therefore, using mesoporous materials loaded with iron nanoparticles is an alternative for fertilizing crops and stimulating tomato plants’ growth and development.

According to the results obtained by XRD and TEM, it was observed that the Fe_3_O_4_@MCM-48 system is a nanomaterial with iron nanoparticles within its pores, which were released by diffusion in controlled quantities, increasing the availability of the mineral and consequently the consumption of the nutrient by the plant.

### 3.2. Impact of Fe_3_O_4_@MCM-48 Nanocomposite on Tomato Plants

One of the main problems of conventional fertilizers in agricultural crops is poor fertilization, due to the formation of insoluble molecules, which also causes strong environmental contamination in soil, water, and air. Currently, the use of nanometric materials as nutrient-carrying systems is an alternative to reduce conventional fertilization problems, because this type of material can release nutrients in a controlled manner, leaving only the nutrients necessary for the plant available, in each one of the growth stages, which significantly reduces the use of conventional agrochemicals and therefore environmental pollution [4].

Fe is a micronutrient that participates in metabolic processes like DNA synthesis, respiration, and photosynthesis, in various biochemical pathways, since it is a component of vital enzymes (chain cytochromes and electron transport). It is a key element in the synthesis of chlorophyll and the maintenance of chloroplast structures and functions, in addition to interacting with various proteins such as ferredoxin and superoxide dismutase [33].

In calcareous soils, with a pH value from neutral to alkaline, iron presents formations of insoluble molecules that are difficult to absorb, limiting its availability, while nanocomposites, when used as nutrient-carrying systems, present some advantages, such as their nanometric size, high surface area, high solubility, and reactivity, to mention a few, which allow them to be easily absorbed and mobilized inside the plant [34]. This has several beneficial impacts, loss of nutrient leaching from the root zone is reduced and thus reduces environmental pollution. It controls the conversion of nutrients from usable to unusable/undesirable forms. Also, the toxic accumulation of soluble salts in the root zone is low, and the slow nutrient release feature improves nutrient uptake by the plant [35]. Interestingly, in this work, no differences were observed between the treatments with Fe_3_O_4_@MCM-48 nanocomposites and the control (ferric chloride) (Figure 3). This clearly indicates that the nanocomposites can completely substitute a conventional iron-based fertilizer—in this case, ferric chloride was used—even though the amount of iron in the nanocomposites is less than that provided by the control. Perhaps most relevantly, MCM-48 (without Fe) was able to provide Fe to the plant (Figure 6), which helped the plant to develop normally (Figure 3). This is probably due to the fact that the work was carried out in soilless culture conditions since a mixture of substrates (peat moss and perlite) was used. In addition, the pH of the nutrient solution was always adjusted to 6.5 to ensure the availability of all elements including Fe. Although Fe was not applied in the nutrient solution, the plants were able to obtain it from the irrigation water with the help of MCM-48. In addition, the development time of the crop was only 100 days, so it is likely that in a complete development cycle (more than six months), differences between treatments could be observed more clearly. Another condition in which differences between treatments could be observed is the development of the experiment in calcareous soil with alkaline pH.

Some studies show that nanoparticles act as elicitors due to interaction with plant cells and can also function as cofactors, micronutrients, antioxidants, and toxins [36]. However, these interactions strongly depend on the nanoparticle characteristics, concentration, and exposure medium. Consumption, translocation, and cellular response will depend on the particular characteristics of nanoparticles and the part of the plant that will be exposed to the material. In the particular case of this research, the Fe_3_O_4_@MCM-48 nanocomposite was applied directly to the roots of tomato plants. Subsequently, magnetite nanoparticles were released from the carrier through a diffusion process mediated by the application of the irrigation solution. The roots consumed these nanoparticles and ascended to the foliage through transport mechanisms, and apoplastic and symplastic pathways [36]. Subsequently, the nanoparticles cross the Casparian strip and move via the transpiration stream. The internalization and interaction with cells and cellular organisms have been studied to enhance the use of nanoparticles. However, endocytosis is considered the most common route of internalization of nanoparticles [36]. Afterward, the plant defense mechanism was activated as part of its natural functioning to neutralize reactive oxygen species produced by the nanoparticles. However, the consumption of magnetite nanoparticles did not cause an overproduction of ROS in tomato plants, Lu et al. [37], indicate that photosynthetic pigments in leaves are affected by the presence of iron nanoparticles, contrary to this work, where the content of photosynthetic pigments increased (Figure 4A–E). The above could be due to the fact that iron at nanometric sizes has a high surface area, which facilitates its absorption through the root and its transport throughout a plant, inducing a greater accumulation of photosynthetic pigments in the leaves. This is in accordance with what was reported by Rout and Sahoo [33], who describe that approximately 80% of the iron absorbed by a plant is found in cells that perform photosynthesis and participate in the electron transport system, this is because it is indispensable for the biosynthesis of cytochromes and other heme molecules.

On the other hand, the literature reports that high production of photosynthetic pigments in crops fertilized with magnetite demonstrates the impact on the plant when it is in contact with nanomaterials, which suggests that derived from this, nanoparticles induce growth and crop development [38].

In this same Figure 4A–E, it is also observed that the photosynthetic pigments of the control treatment (ferric chloride) were affected by the drench application of ferric chloride, while MCM-48 presents high contents of the same. This indicates that the increase in photosynthetic pigments is due to the incorporation of silicon into the nanomaterial. It has been shown that the application of silicon (Na_2_SiO_3_) alone is capable of increasing the content of chlorophylls and carotenoids in wheat plants [39]. The authors suggest that the observed effect may be linked to the decrease in reactive oxygen species in plant cells. In this sense, it has been proposed that silicon has the ability to protect photosynthetic pigments in plants and reduce damage to the structure of chloroplasts, as well as intervene in the increase in the expression of genes related to photosynthesis [40]. Additionally, the authors mention that under the influence of silicon, micro and macronutrients regulate photosynthetic processes, as well as phytohormones can also influence photosynthetic processes. Derived from this, it is possible to hypothesize that in this work, the greatest influence on photosynthetic pigments was due to the presence of silicon in the nanocomposite used. While it is true that Fe is fundamental on its own, the interaction of Fe with silicon seems to be more beneficial to plants. Although the implications of the plant–silica interaction are not fully understood, some studies indicate that nanometric silica is able to penetrate the cell wall and reach the plasma membrane, then accumulate, causing changes at various cellular and physiological levels of the plant, which causes an increase in plant growth, protein yield, and chlorophyll content in various plants, including tomatoes [6,41].

As mentioned above, one of the important roles of iron is its participation in plant photosynthesis. According to reports by Hussein et al. [42] iron nanoparticles increase the photosynthetic activity and therefore the production of sugars, which in turn leads to a higher concentration of sugars and TSS. This is in agreement with the results obtained here (Figure 5C), where an increase in TSS content is observed in a dependent manner to Fe content in the treatments, where the treatment with 50% Fe (50/50) presents the highest TSS content in tomato fruits. This could be due to the fact that iron nanoparticles, when absorbed, participate in the metabolic processes of plants, improving the fruit quality, particularly TSS and total sugar [43]. The above is a clear indication of the modification in the biological metabolism of the plant by the interaction with iron nanoparticles [38]. Although there are several publications on the positive effects of nanoparticles and/or nanomaterials on agronomical and physicochemical parameters in plants [16,34,44,45], in this research, no statistically significant differences were observed in agronomical parameters; however, at the biochemical level, positive impacts were observed.

## 4. Materials and Methods

### 4.1. Materials

Tetraethylorthosilicate (TEOS 98%), cetyltrimethiammonium bromide (CTAB 98%), ethanol (99.8%), ammonium hydroxide (NH_4_OH 29%), and ferric chloride hexahydrate (FeCl_3_*6H_2_O) were obtained from Sigma-Aldrich (St. Louis, MO, USA).

### 4.2. Mesoporous Silica Synthesis (MCM-48)

The synthesis of MCM-48 was performed according to the method of Meléndez-Ortiz [46]. In total, 2.6 g of CTAB was added to 120 mL of distilled H_2_O and 50 mL of ethanol under constant stirring. Once a homogeneous solution was obtained, 10 mL of NH_4_OH was added. After that, 2 mL of TEOS was poured into the above solution under vigorous stirring for 16 h at room temperature. The solid product was obtained after filtering and subsequently dried at 70 °C for 24 h. The CTAB was removed from the inorganic material by sintering the sample at 540 °C for 9 h.

### 4.3. Synthesis of Fe_3_O_4_@MCM-48

The synthesis of Fe_3_O_4_@MCM-48 was performed according to the method of Meléndez-Ortiz [46]. 169.26 mg of FeCl_3_.6H_2_O were added to 120 of distilled H_2_O under stirring, then 2.6 g of CTAB and 50 mL of ethanol were added. After a homogeneous solution was obtained, 10 mL of NH_4_OH was added. Finally, 2 mL of TEOS was added to the above solution under vigorous stirring for 16 h at room temperature. The solid product was obtained after filtering and subsequently dried at 70 °C for 24 h. The sample obtained was sintered at 540 °C for 9 h to remove the CTAB and form the iron oxide. Different percentages of Fe_3_O_4_ were incorporated into the MCM-48, as shown in Table 2.

### 4.4. Characterization of MCM-48

Through X-ray diffraction (XRD) was determined the crystalline structure of the materials obtained, for this, a Bruker D8 Advance diffractometer (Bruker, Billerica, MA, USA) was used, with Cu Kα radiation (40 kV, 44 mA) in a range of 2–80° measurement on the 2θ scale with a sweep speed of 0.02°/s. The quantification of Fe in the MCM-48 was carried out using ICP spectrometry (thermo-scientific iCAP 7000 SERIES spectrometer, Waltham, MA, USA). The morphology and sample size were examined through an FEI Titan 80–300 high-resolution transmission electron microscope (HRTEM) (Hillsboro, OR, USA).

### 4.5. Crop Management

Seeds of hybrid tomato of indeterminate growth (El CID F1) type saladette (*Solanum lycopersicum* L.) were germinated in a peat moss and perlite mixture as substrate in a 1:1 (*v*/*v*) ratio and grew for 32 days. After this, the transplant was carried out in 10 L black polyethylene bags with the substrate mixture. Crop nutrition was carried out using a Steiner nutrient solution [47] at each irrigation, dosed according to the growth stage of the plant and its demand, i.e., a concentration of 25% was applied from transplanting, at the beginning of flowering it was increased to 50%, 75% when fruit set began, and 100% from fruit filling. The pH of the nutrient solution was maintained at 6.5. The Steiner solution contained the following fertilizers: Ca(NO_3_)_2_ (1060 mg L^−1^), MgSO_4_ (487 mg L^−1^), KNO_3_ (71 mg L^−1^), K_2_SO_4_ (347 mg L^−1^), KH_2_PO_4_ (211 mg L^−1^), the micronutrients were added as chelates. Fe was not added to the nutrient solution. The plants were managed with a single stem with Dutch-type stakes, the planting density was 3 plants per m^2^, and the crop was maintained for 100 days.

### 4.6. Application of Treatments

Solutions with the different concentrations of Fe_3_O_4_ and MCM-48 were prepared as presented in Table 2; ferric chloride and pure MCM-48 were also used as controls. Then, 500 mg of each nanocomposite of Table 2 were suspended in 150 mL of distilled water and sonicated for 10 min in a BK-2000 ultrasonic cleaner, and 10 mL of solution with 33.33 mg of the nanocomposite was taken and applied via drench to each plant. In the case of the controls, 500 mg of ferric chloride (control) or 500 mg of pure MCM-48 (without Fe) were used. The applications began seven days after the transplant and were repeated at 30-day intervals until the completion of 3 applications.

### 4.7. Determination of Growth and Biomass

Once the evaluation period ended, the following analyses of the plants were carried out: stem diameter, plant height, number of leaves, number of clusters, and for the total biomass, the difference between the fresh weight (leaves, stem, and root), and dry weight of each variable was considered.

### 4.8. Biochemical Variables Evaluated

In foliar tissue, the contents of chlorophyll *a*, *b*, and total (mg 100 g^−1^ DW) were determined according to the method of Nagata and Yamashita [48]. The lyophilized sample (10 mg) was mixed with 2 mL of hexane:acetone (3:2). Subsequently the samples were subjected to an ultrasonic bath for 5 min. They were then centrifuged at 15,000× *g* for 10 min at 4 °C. The supernatant was removed and the absorbance was read at 645 and 663 nm using a UV–Vis spectrophotometer (UNICO, Model UV2150, Dayton, NJ, USA). The obtained values were used in Equations (1) and (2) to calculate the chlorophyll *a* and chlorophyll *b* content.

(1) $$Chlorophyll\;a=25.38*A_{663}+3.64*A_{645}$$

(2) $$Chlorophyll\;b=30.38*A_{645}-6.58*A_{663}$$

The contents of lycopene (mg 100 g^−1^ DW) and β-carotene (mg 100 g^−1^ DW) were determined for both leaf tissue and fruits, following the method of Nagata and Yamashita [48]. The lyophilized sample (10 mg) was mixed with 2 mL of hexane:acetone (3:2). Subsequently the samples were subjected to an ultrasonic bath for 5 min. They were then centrifuged at 15,000× *g* for 10 min at 4 °C. The supernatant was removed and the absorbance was read at 453, 505, 645, and 663 nm using a UV–Vis spectrophotometer (UNICO, Model UV2150, Dayton, NJ, USA). The obtained values were used in Equations (3) and (4) to calculate the lycopene and β-carotene content.

(3) $$Lycopene=-0.0458*A_{663}+0.204*A_{645}+0.372*A_{505}-0.0806*A_{453}$$

(4) $$\beta -carotene=0.216*A_{663}-1.22*A_{645}-0.304*A_{505}+0.452*A_{453}$$

### 4.9. Physicochemical Parameters of the Fruits

Hydrogen potential (pH), total soluble solids (TTS), titratable acidity (AT), and fruit firmness were determined. For this, fruits were collected at a “full red” level of maturity, verifying that they were not physically damaged and that they were uniform. Three fruits per treatment were selected, each one from a different plant, and were washed, weighed, and measured (polar and equatorial diameter). Through a digital combo pH/conductivity tester (HI 98130, Hanna Instruments, Woonsocket, RI, USA) hydrogen potential (pH) and electrical conductivity (EC) were determined. Total soluble solids (TSS) were determined using a digital refractometer (ATAGO, MASTER-100H model, Bellevue, WA, USA). For the determination of fruit firmness, a manual penetrometer (Wagner Instruments, FDK 20 model, Greenwich, CT, USA) was used. The fruit firmness was measured in three different points of the fruit and reported as the average. In addition, the color parameters L*, a*, and b* (CIELAB) were determined with a portable color analyzer (BELEY, model WR10QC, FRU, Guangzhou, China).

### 4.10. Statistical Analysis

The experiment was established considering a randomized complete block design, with seven treatments and three repetitions each, the experimental unit was made up of four plants. Data analysis was performed using analysis of variance and Fisher’s least significant difference mean comparison test (*p* ≤ 0.05). The statistical package InfoStat version 2021 was used.

## 5. Conclusions

This work shows the use of the obtained Fe_3_O_4_@MCM-48 nanocomposite as nano fertilizer to stimulate the growth and development of tomato plants. The nano fertilizer could release iron nanoparticles through diffusion, providing the plant with sufficient quantities of the micronutrient for its nutrition, avoiding common iron chlorosis problems. Therefore, it is possible to use a nanofertilizer as a source of Fe for plants and to replace common sources.

In the agronomic variables, it was demonstrated that nanocomposites can completely substitute a conventional fertilizer (in this case, ferric chloride), even though the amount of Fe applied through these was lower. However, no differences were observed between the Fe_3_O_4_@MCM-48 nanocomposites and pure MCM-48, this could be due to the fact that the work was developed in soilless culture and the pH of the nutrient solution was regulated, which favored the availability of Fe. In addition, the crop cycle was short, only 100 days from transplanting, so the demand for Fe was not so high. For these reasons, it is advisable to carry out new experiments considering the development of plants in calcareous soils with alkaline pH, and for a crop development period of at least six months.

The most relevant results show increased photosynthetic pigments (up to 67%), lycopene (up to 55%), and β-carotene (23%) in leaves caused by the Fe_3_O_4_@MCM-48 nanocomposite and MCM-48 pure presence compared to the control (ferric chloride), demonstrating the positive impact of the nanocomposites used.

It is possible that this Fe-based nanofertilizer could be potentially effective under Fe-limiting conditions for the plant. However, it is necessary to carry out more studies in growing conditions in calcareous or alkaline soils, where it is more difficult to have Fe available for plants.

## Figures and Tables

**Table 1:** Amount of iron available to stimulate growth in tomato plants.

Ratio MCM-48/Fe_3_O_4_	Fe_3_O_4_ (ppm)
90/10	46,380
80/20	106,550
70/30	161,730
60/40	203,610
50/50	284,330

**Table 2:** Quantity of reagents used for the different MCM-48/Fe_3_O_4_.

Samples/Reagents	FeCl_3_*6H_2_O (mg)	CTAB (g)	Water (mL)	Ethanol (mL)	NH_4_OH (mL)	TEOS (mL)
MCM-48 90/10 Fe_3_O_4_	169.26	2.60	120.00	50.00	10.0	2.0
MCM-48 80/20 Fe_3_O_4_	338.53	2.31	106.66	44.44	8.9	1.8
MCM-48 70/30 Fe_3_O_4_	507.79	2.02	99.33	38.38	7.8	1.6
MCM-48 60/40 Fe_3_O_4_	677.06	1.73	80.00	33.33	6.7	1.3
MCM-48 50/50 Fe_3_O_4_	846.32	1.44	66.66	27.77	5.6	1.1

## Data Availability

Data are contained within the article and Supplementary Materials.

## Supplementary Materials

The following supporting information can be downloaded at: https://www.mdpi.com/article/10.3390/plants14030405/s1. Table S1. Coefficient of variation and analysis of variance of agronomic variables. Table S2. Coefficient of variation and analysis of variance of leaf pigments, and fruit pigments and color. Table S3. Coefficient of variation and analysis of variance of agronomic variables. Table S4. Coefficient of variation and analysis of variance of physico-chemical parameters of tomato fruits.
