# Supplementary material for: Synthesis of Fe3O4@MCM-48 as Nano Fertilizer for Growth Stimulation in Tomato Plants
Source: Plants (Basel). 2025 Jan 29;14(3):405. doi: 10.3390/plants14030405 (PMC11820872; doi:10.3390/plants14030405)
Supplement: Supplementary file 1 [file plants-14-00405-s001.zip › plants-3364250-supplementary.pdf]

Table S1. Coefficient of variation and analysis of variance of agronomic variables.

Variable	CV (%)	SC_T	GL	CM	F	p-value
Dry biomass of plant	11.17	1122.59	6	187.10	0.81	0.5785
Fresh biomass of plant	10.02	52600.58	6	8766.76	1.32	0.3193
Dry biomass of leaves	9.32	96.44	6	16.07	1.23	0.3553
Fresh biomass of leaves	5.16	4981.48	6	830.25	3.56	0.0291
Fruit yield	9.87	318799.04	6	53133.17	0.26	0.9474

Table S2. Coefficient of variation and analysis of variance of leaf pigments, and fruit pigments and color.

Variable	CV (%)	SC_T	GL	CM	F	p-value
β-carotene (leaf)	6.16	0.01	6	2.5E-03	3.25	0.0324
Lycopene (leaf)	11.37	4.1E-03	6	6.8E-04	3.55	0.0294
Total chlorophyll (leaf)	13.47	1192.99	6	198.83	3.68	0.0261
Chlorophyll a (leaf)	13.66	783.61	6	130.60	3.58	0.0286
Chlorophyll b (leaf)	12.96	43.35	6	7.23	4.00	0.0196
L* (fruit)	2.88	1.88	6	0.31	0.23	0.9587
a* (fruit)	2.53	1.97	6	0.33	1.06	0.4381
b* (fruit)	4.43	12.26	6	2.04	1.51	0.2565
β-carotene (fruit)	17.08	8.3E-04	6	1.4E-04	0.20	0.9703
Lycopene (fruit)	19.82	0.02	6	2.5E-03	0.33	0.9095

Table S3. Coefficient of variation and analysis of variance of agronomic variables.

Variable	CV (%)	SC_T	GL	CM	F	p-value
Electrical conductivity	12.02	0.55	6	0.09	0.56	0.7553
pH	1.39	0.02	6	3.4E-03	0.83	0.5672
TSS	3.14	1.06	6	0.18	9.68	0.0005
Firmness	11.50	0.11	6	0.02	0.21	0.9665
Equatorial diameter	2.08	42.50	6	7.08	4.96	0.0090
Polar diameter	2.48	38.13	6	6.36	1.68	0.2080
Fruit weight	5.44	533.31	6	88.89	1.42	0.2841

Table S4. Coefficient of variation and analysis of variance of physico-chemical parameters of tomato fruits.

Variable	CV (%)	SC_T	GL	CM	F	p-value
Iron	25.47	444.48	6	74.08	0.35	0.8993
Silicon	50.43	0.19	6	0.03	0.52	0.7857
